# Early myeloid-derived suppressor cells (HLA-DR^−^/^low^CD33^+^CD16^−^) expanded by granulocyte colony-stimulating factor prevent acute graft-versus-host disease (GVHD) in humanized mouse and might contribute to lower GVHD in patients post allo-HSCT

**DOI:** 10.1186/s13045-019-0710-0

**Published:** 2019-03-18

**Authors:** Ke Wang, Meng Lv, Ying-Jun Chang, Xiang-Yu Zhao, Xiao-Su Zhao, Yuan-Yuan Zhang, Yu-Qian Sun, Zhi-Dong Wang, Pan Suo, Yang Zhou, Dan Liu, Shu-Zhen Zhai, Yan Hong, Yu Wang, Xiao-Hui Zhang, Lan-Ping Xu, Kai-Yan Liu, Xiao-Jun Huang

**Affiliations:** 1 Peking University People’s Hospital, Peking University Institute of Hematology,Beijing Key Laboratory of Hematopoietic Stem Cell Transplantation, National Clinical Research Center for Hematologic Disease, No 11 Xizhimen South Street, Beijing, 100044 China; 2 Center for Life Sciences, Academy for Advanced Interdisciplinary Studies, Peking University, No.5 Yiheyuan Road, Beijing, 100871 China

**Keywords:** Myeloid-derived suppressor cells, Granulocyte colony-stimulating factor, Acute graft-versus-host disease, Humanized mouse, Allogeneic hematopoietic stem cell transplantation

## Abstract

**Introduction:**

Myeloid-derived suppressor cells (MDSCs) are proposed to control graft-versus-host disease (GVHD) in allogeneic hematopoietic stem cell transplantation (allo-HSCT). However, the definition of human MDSCs has not yet reached consensus, and the mechanism of MDSCs to control GVHD remains unclear.

**Methods:**

Immature myeloid cells (HLA-DR^−/low^CD33^+^CD16^−^) were tested before and after granulocyte colony-stimulating factor (G-CSF) administration in healthy donor and isolated for suppression assays and co-culture with T cells in vitro. Isolated cells were infused in humanized mice for a xenogeneic model of acute GVHD. One hundred allo-HSCT recipients were enrolled prospectively to assess the role of HLA-DR^−/low^CD33^+^CD16^−^ cells in grafts on the occurrence of acute GVHD.

**Results:**

In the present study, G-CSF mobilized HLA-DR^−/low^CD33^+^CD16^−^ cells with immunosuppressive properties in donor peripheral blood. These cells contained more interleukin-10^+^ and transforming growth factor-beta (TGF-β)^+^ cells after G-CSF administration and inhibited the proliferation of autologous donor T cells in a TGF-β-dependent manner. Meanwhile, these immature myeloid cells promoted regulatory T cell expansion and induced Th2 differentiation. Importantly, these cells prevented acute GVHD in a humanized mouse model. Moreover, clinical cohort results showed that the number of HLA-DR^−/low^CD33^+^CD16^−^ cells in the donor graft was the only independent risk factor inversely correlated with the incidence of grade II–IV acute GVHD in the recipients (HR 0.388, 95% CI 0.158–0.954, *p* = 0.039).

**Conclusion:**

HLA-DR^−/low^CD33^+^CD16^−^ cells represent functional MDSCs that may control acute GVHD in allo-HSCT.

**Electronic supplementary material:**

The online version of this article (10.1186/s13045-019-0710-0) contains supplementary material, which is available to authorized users.

## Introduction

Allogeneic hematopoietic stem cell transplantation (allo-HSCT) is an effective treatment for patients with most hematological diseases [1]. However, the applications of allo-HSCT are limited by lethal complications, including graft-versus-host disease (GVHD). The increased understanding of the regulatory cells and molecular pathways involved in limiting pathogenic immune responses offers the opportunity for promoting GVHD-relapse-free survival (GRFS), aiming to replace non-specific GVHD control with pharmacological immune suppression [2–5].

Extensive research has shown that myeloid-derived suppressor cells (MDSCs) have emerged as major immunosuppressive cells in cancer, infection, chronic inflammation, and autoimmune disease, which suggest that MDSCs may help to control GVHD [6, 7]. However, the phenotypic, morphological, and functional heterogeneity of MDSCs generates confusion in the investigation and analysis of their roles in immune responses as MDSCs represent a heterogeneous population of immature myeloid cells and are defined by their ability to suppress T cells in vitro [8, 9]. In humans, MDSCs most commonly express the common myeloid marker, CD33 [10], but lack the expression of markers of mature myeloid and lymphoid cells as well as the MHC class-II molecule, HLA-DR [11, 12]. Lin^−^ (including CD3, CD19, and CD56) CD14-CD15-HLA-DR^−^CD33^+^ cells contain mixed groups of MDSCs comprising more immature progenitors (also named early MDSCs, eMDSCs). Furthermore, two other subpopulations with more differentiated surface makers, namely, CD15+ or CD14+, are divided into two groups as follows: G-MDSCs (PMN-MDSCs) and M-MDSCs [8]. However, the definitions of these subsets have not yet reached consensus, and it remains unclear if MDSCs control GVHD in vivo.

Though granulocyte colony-stimulating factor (G-CSF) has been routinely used to mobilize stem cells to the peripheral blood from healthy donors, it is also recognized as a novel mediator of T cell tolerance [13, 14]. Increasing evidences suggest that G-CSF affects different immune cells [13, 15], which modulate T cell response directly by inducing Th2 differentiation [16, 17] or mobilizing functional regulatory T cells [18], or indirectly through monocytes [19–21], DC [22], and neutrophils [23] to prevent GVHD. Previous results have suggested that G-CSF-mobilized donor cells also contain MDSCs, which are closely associated with GVHD in allo-HSCT [24]. Antonio et al. reported that systemic treatment with G-CSF induces expansion of myeloid cells displaying the phenotype of M-MDSCs (Lin^low/neg^HLA-DR^−^CD11b^+^CD33^+^CD14^+^), which is the only graft parameter to predict acute GVHD (aGVHD) [25]. Lv showed that monocytic MDSCs (Lin^−^HLA-DR^−/low^CD33^+^CD11b^+^CD14^+^CD15^dim^CD16^−^) and promyelocytic MDSCs (Lin^−^HLA-DR^−/low^CD33^+^CD11b^−/low^CD14^−^CD15^−^CD16^−^) in the graft are correlated with the incidence of cGVHD without significant influence on relapse and survival [26]. Fan demonstrated that higher frequency of Lin^low/neg^HLA-DR^−^CD33^+^CD11b^+^ MDSCs in the G-CSF-primed bone marrow (G-BM) than in the G-CSF peripheral blood stem cell (G-PBSC) harvest grafts leads to a better GRFS and less GVHD [27]. In contrast, the frequency of CD14^+^HLA-DR^low/neg^M-MDSCs is significantly increased in the peripheral blood after allo-HSCT, especially in patients with acute graft-versus-host disease [28]. Though clinical relevance between MDSCs and GVHD has been observed in humans, there is no data available to identify if human MDSCs prevent GVHD in vivo and there was limited data about the eMDSCs in allo-HSCT. In addition, as positive CD16 represents the relatively mature marker of myeloid cells, whether negative CD16 could better define MDSCs remains to be explored [26, 29].

Based on a humanized mouse model and a prospective cohort study, the present study demonstrated that HLA-DR^−/low^CD33^+^CD16^−^ cells expanded by G-CSF represent subsets of more immature progenitors and immune regulatory properties in donor PBSC have the immunosuppressive potential of eMDSCs, which suppress aGVHD in the allo-HSCT settings.

## Materials and methods

### Experimental model

NOD-SCID-IL-2Rg−/− mice (NSG mice) were purchased from Vitalstar. All mice were bred and housed in a specific pathogen-free facility in microisolator cages and used at 8 to 12 weeks of age in protocols approved by the local Ethical Committee. Each experimental group included six to eight mice, and the experiment is repeated twice.

### Donor PB and BM samples

This study was approved by the Ethics Committee of Peking University People’s Hospital, Beijing, in accordance with the Declaration of Helsinki. Healthy donors were enrolled from the related donors of patients who had undergone either haplo-identical or HLA-matched allogeneic blood and marrow transplantation at the Peking University People’s Hospital between April 2017 and May 2018. Peripheral blood and BM samples (10 ml in EDTA tube) from recombinant human G-CSF (rhG-CSF)-non-treated or recombinant human G-CSF-treated (filgrastim, 5 μg/kg/day, five consecutive days) healthy allogeneic donors (randomly selected at Peking University People’s Hospital in Beijing) were collected after informed consent was obtained.

### Flow cytometry

Different MDSC types in donor PB and BM samples were analyzed using flow cytometry (FACS Canto II, BD Biosciences) with the following antibodies: CD45, CD3, CD19, CD56, HLA-DR, CD11b, CD33, CD14, CD15, and CD16 (eBioscience or BD Bioscience) (Table 1).

**Table 1:** Definition of MDSCs

Human MDSCs	Phenotype
Total MDSC	Lin^−^HLA-DR^−/low^CD33^+^
PMN-MDSC (G-MDSC)	Lin^−^HLA-DR^−/low^CD33^+^CD11b^+^CD14^−^CD15^+^
M-MDSC	Lin^−^HLA-DR^−/low^CD33^+^CD11b^+^CD14^+^CD15^−^
eMDSCs	Lin^−^HLA-DR^−/low^CD33^+^CD16^−^
Lin	CD3, CD19, CD56

### Human HLA-DR^−/low^CD33^+^CD16^−^ isolation

HLA-DR^−/low^CD33^+^CD16^−^ cells were isolated from GPBSC using fluorescence-activated cell sorting (FACS)—sorted (FACS Aria II, BD Biosciences) with antibodies of HLA-DR, CD33, and CD16 (eBioscience or BD Bioscience) according to the manufacturer’s instructions, which included one step of positive selection for CD33^+^ cells and two steps of negative selection for HLA-DR^−^ and CD16^−^ cells.

### Morphology

HLA-DR^−/low^CD33^+^CD16^−^ morphology was studied on cytospins after May-Grünwald-Giemsa staining. Photographs were taken using × 1000 magnification with a smart V350Df digital camera mounted on an OLYMPUS CX31 microscope.

### T cell proliferation and suppression assays

T cells from GPBSC were purified by positive selection using CD3 cell isolation via flow cytometry. Purified CD3^+^ T lymphocytes were labeled with 5 μM 5,6-carboxy-fluorescein diacetate succinimidyl ester (CFSE) (eBioscience) and thereafter were co-cultured with isolated HLA-DR^−/low^CD33^+^CD16^−^ at a HLA-DR^−/low^CD33^+^CD16^−^ to CD3^+^ T cell ratio of 0.25:1 to 2:1 in the presence of anti-CD3/CD28 beads (Thermo) in RPMI 1640 (Biological Industries) supplemented with 10% FBS, penicillin/streptomycin, and 2 mM l-glutamine. 10^5^ T cells were seeded in 96-well U-bottom plates. After 4 days, cells were harvested for flow cytometry analysis. To neutralize IL-10 and TGF-β, blocking antibodies were used at 10 to 20 μg/ml concentrations (blocking antibody for IL-10 and TGF-β from R&D Systems). To inhibit arginase activity, nor-NOHA (Calbiochem) was used at a concentration of 300 μM. For the inhibition of iNOS, l-NMMA (Sigma) was used at a concentration of 300 μM. To inhibit IDO, indoximod (NLG8189) (Selleck) was used at a concentration of 500 μM. To inhibit Cox2, NS398 (Selleck) was used at a concentration of 10 μM. All the in vitro experiment was repeated at least three times.

### T helper cells detected after co-culture with MDSCs

T cells from G-PBSC were sorted via flow cytometry. Purified CD3^+^ T lymphocytes were co-cultured with isolated HLA-DR^−/low^CD33^+^CD16^−^ at a HLA-DR^−/low^CD33^+^CD16^−^ to CD3^+^ T cell ratio of 1:1 in the presence of anti-CD3/CD28 beads (Thermo) in RPMI 1640 (Biological Industries) supplemented with 10% FBS, penicillin/streptomycin, and 2 mM l-glutamine. Four days later, the percentages of Th1 (CD4^+^IFNγ^+^), Th2 (CD4^+^IL-4^+^), and Th17 (CD4^+^IL-17A^+^) were detected via flow cytometry, then the fold changes of Th2/Th1 and Th2/(Th1+Th17) were calculated. Human Th1/Th2/Th17 Phenotyping Kit (BD Pharmingen) was used.

### Regulatory T cell (Treg) detected after co-culture with MDSCs

T cells from G-PBSC were sorted via flow cytometry. Purified CD3^+^ T lymphocytes were co-cultured with isolated HLA-DR^−/low^CD33^+^CD16^−^ at a ratio of 1:1 in RPMI 1640 (Biological Industries) supplemented with 10% FBS, penicillin/streptomycin, and 2 mM l-glutamine. Four days later, the percentage of Treg (CD4^+^CD25^+^FoxP3^+^) was detected via flow cytometry.

### Xenogeneic model of graft-versus-host disease

NSG mice were sublethally irradiated using 180-cGy total body irradiation by X-ray on day − 1, followed by the intravenous infusion in the caudal vein of 5 × 10^6^ GPBSC and different dose of HLA-DR^−/low^CD33^+^CD16^−^ on day 0. Control groups were transplanted with GPBSC alone. Engraftment of human white blood cell was detected from peripheral blood samples of mice at 7 days, 14 days, and 21 days after transplantation. Engraftment of human eMDSC was detected in the peripheral blood, spleen, and bone marrow of humanized mice at 14 days and 21 days after transplantation. Human Treg (CD4^+^CD25^+^Foxp3^+^), Th1 (CD4^+^T-bet^+^), Th2 (CD4^+^GATA3^+^), Th17 (CD4^+^RORγt^+^), and the cytokine levels were detected from peripheral blood samples of mice at 21 days after transplantation. Levels of cytokines were assessed with pre-designed sets of Luminex immunoassays from eBioscience (for all cytokines measured in supernatants [Human ProcartaPlex Panel #EPX180-12165-901] according to the recommended protocols, using Magpix detection platform (Luminex Corp., Austin, TX) and xPonent software (Luminex). Mice were monitored for survival, weight, and acute GVHD score three times a week. The clinical scoring system was based on six parameters: weight loss, posture, activity, fur texture, skin integrity, and diarrhea. A severity scale of 0 to 1 was used for each parameter, with a maximum score of 6. Mice that had died or had been sacrificed at 1.5 months after HSCT and the tissues were kept in 4% paraformaldehyde. Tissues from GVHD target organs (liver, intestine, and skin) were embedded in paraffin, sectioned, and stained with hematoxylin, eosin, and Safran. Photographs were taken using × 200 or × 400 magnifications with Basler Microscopy ace 2.3 MP Mono camera mounted on an OLYMPUS BX43 microscope.

### Transplant procedure of allo-HSCT

The conditioning therapy for patients receiving allo-HSCT from matched sibling donors (MSDs) for acute myeloid leukemia (AML), acute lymphoid leukemia (ALL), and myelodysplastic syndromes (MDS) is as follows: intravenous cytarabine (2 g/m^2^/day) on day − 9, intravenous busulfan (3.2 mg/kg/day) on days − 8 to − 6, intravenous cyclophosphamide (1.8 g/m^2^/day) on days − 5 to − 4, and oral methyl chloride hexamethylene urea nitrate (Me-CCNU; 250 mg/m^2^/day) on day − 3. The GVHD prophylaxis regimen consisted post-transplant cyclosporine A, mycophenolate mofetil, and short-term methotrexate [30–32].

The conditioning therapy for patients receiving allo-HSCT from HLA haplo-identical donors for AML, ALL, and MDS is as follows: intravenous cytarabine (4 g/m^2^/day) on days − 10 to − 9 and intravenous ATG (2.5 mg/kg/day; Sang Stat, Lyon, France) on days − 5 to − 2, while the application of busulfan, cyclophosphamide, Me-CCNU, and post-transplant GVHD prophylaxis was similar to allo-HSCT from MSDs [30–32].

The conditioning therapy for patients receiving allo-HSCT from MSDs for severe aplastic anemia (SAA) is as follows: intravenous ATG (2.5 mg/kg/day; Sang Stat, Lyon, France) on days − 5 to − 2 and intravenous cyclophosphamide (50 mg/kg/day) on days − 5 to − 2. Intravenous busulfan (3.2 mg/kg/day) on days − 7 to − 6 was added for haplo-HSCT recipients of SAA. Post-transplant GVHD prophylaxis was similar to allo-HSCT for acute leukemia [33, 34].

### Survival analysis of allo-HSCT recipients

The risk stratification for recipients of hematological malignancies is followed by Disease Risk Index (DRI) [35]. The survival functions were estimated by the Kaplan-Meier method using the log-rank test with asymmetric 95% confidence intervals, and the cumulative incidences of aGVHD or cGvHD and TRM/Relapse were calculated using competing risks with 95% confidence intervals. Diagnosis of GVHD was also evaluated with classic and the National Institutes of Health (NIH) consensus criteria [36, 37]. Univariate probabilities of survival were calculated using a left-truncated approach. A bivariable analysis was applied to check with two-way interactions between variables (Spearman correlation for non-normally distributed values; Pearson correlation for normally distributed values). All variables included in the multivariable analysis were tested with time-dependent covariates and constructed in a proportional hazards (Cox) model if PH ≥ 0.05 or a Cox w/Time-Dep Cov model if PH < 0.05, which included patient age, sex, donor-patient sex mismatch, graft components, etc. Variables included in the multivariate analysis were selected by backward elimination process with a criterion of *p* < 0.10 for retention.

## Results

### HLA-DR^−/low^CD33^+^CD16^−^ cells expanded in G-CSF-mobilized PBSC suppressed T lymphocyte proliferation

**Fig. 1:**
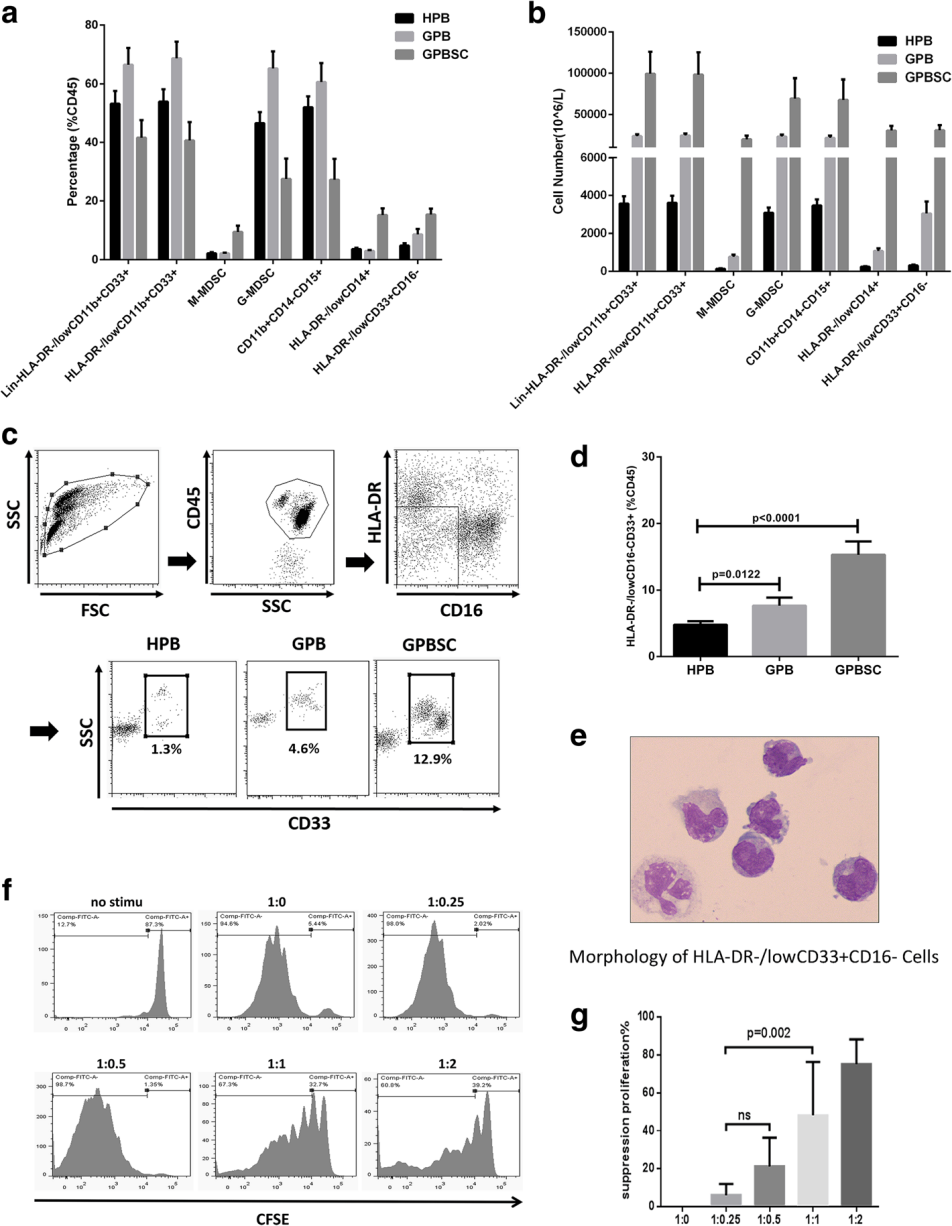
HLA-DR^−/low^CD33^+^CD16^−^ myeloid cells expanded in PB and PBSC after G-CSF administration and exerted a strong immunosuppressive effect on T cell proliferation. **a**, **b** The frequency and cell number of different kind of MDSCs and HLA-DR^−/low^CD33^+^CD16^−^ population in ten donors (H-PB, G-PB, G-PBSC) were analyzed by flow cytometry. **c** Gate strategies of HLA-DR^−/low^CD33^+^CD16^−^: FSC, forward scatter; SSC, side scatter. **d** The proportion of HLA-DR^−/low^CD33^+^CD16^−^ in the CD45^+^ fraction from the ten healthy donor’s HPB, GPB, and GPBSC (medians of the different groups were 4.6%, 6.5%, and 15.5% of total CD45+cells, Mann-Whitney *t* test). **e** May-Grünwald-Giemsa cytospin preparations show morphological features of HLA-DR^−/low^CD33^+^CD16^−^. **f** T cell proliferation was examined using CFSE dilution. HLA-DR^−/low^CD33^+^CD16^−^ and CD3^+^ T cells from the same donor G-PBSC were co-cultured at different ratios for 4 days with anti-CD3/CD28 beads. T cell proliferation was evaluated using CFSE labeling. Unstimulating T cells were negative control. The picture shows the representative results. **g** The percentage of T cells in suppression was shown in different groups. Data was compared using unpaired *t* test (ns, not significant)

To investigate the types of MDSCs or other myeloid cells expanded in the peripheral blood after G-CSF treatment in donors, the frequency and cell number of different MDSCs were analyzed. Total MDSCs (HLA-DR^−/low^CD11b^+^CD33^+^), M-MDSCs (HLA-DR^−/low^CD11b^+^CD33^+^CD14^+^CD15^−^), G-MDSCs (HLA-DR^−/low^CD11b^+^CD33^+^CD14^−^CD15^+^), CD11b^+^CD14^−^CD15^+^ cells (alternative definition of G-MSCSs), HLA-DR^−/low^CD14^+^ cells (alternative definition of M-MSCSs), and HLA-DR^−/low^CD33^+^CD16^−^ cells in the peripheral blood from ten healthy donors before (homeostasis peripheral blood, H-PB) and after G-CSF mobilization (G-CSF-mobilized peripheral blood, G-PB), as well as G-PBSC, were analyzed. The cell numbers and percentage of all MDSCs were significantly increased in G-PB and G-PBSC. M-MDSC population increased in both frequency and absolute number in G-PBSC. HLA-DR^−/low^CD33^+^CD16^−^ population increased in both frequency and absolute number in G-PB and G-PBSC (Fig. 1a, b, d).

May-Grünwald-Giemsa cytospin results showed that the morphological features of HLA-DR^−/low^CD33^+^CD16^−^ cells were similar to those of immature monocyte-like cells (Fig. 1e). The in vitro immune-suppressive activity of the HLA-DR^−/low^CD33^+^CD16^−^ population identified among the G-PBSC was tested. HLA-DR^−/low^CD33^+^CD16^−^ and autologous CD3^+^ T cells were sorted from the G-PBSC of healthy donors using FACS. HLA-DR^−/low^CD33^+^CD16^−^ cells were co-cultured for 4 days with autologous T cells at different ratios (HLA-DR^−/low^CD33^+^CD16^−^: *T* = 0:1 to 1:2) in the presence of T cell stimulators (anti-CD3/CD28 beads). Flow cytometry analysis demonstrated that the HLA-DR^−/low^CD33^+^CD16^−^ population had a strong immunosuppressive effect on T cell proliferation in a cell dose-dependent manner (Fig. 1f, g). Moreover, HLA-DR^−/low^CD33^+^CD16^−^ population demonstrated superior immune-suppressive activity compared with CD33^−^ and HLA-DR^−/low^CD33^+^CD16^+^ fraction (Additional file 1: Figure S1). Meanwhile, the immune-suppressive activity of HLA-DR^−/low^CD33^+^CD16^−^ cells was inferior to M-MDSCs and G-MDSCs population (Additional file 1: Figure S2).

### HLA-DR^−/low^CD33^+^CD16^−^ cell-induced inhibition of T cell proliferation in vitro depends on TGF-β production

**Fig. 2:**
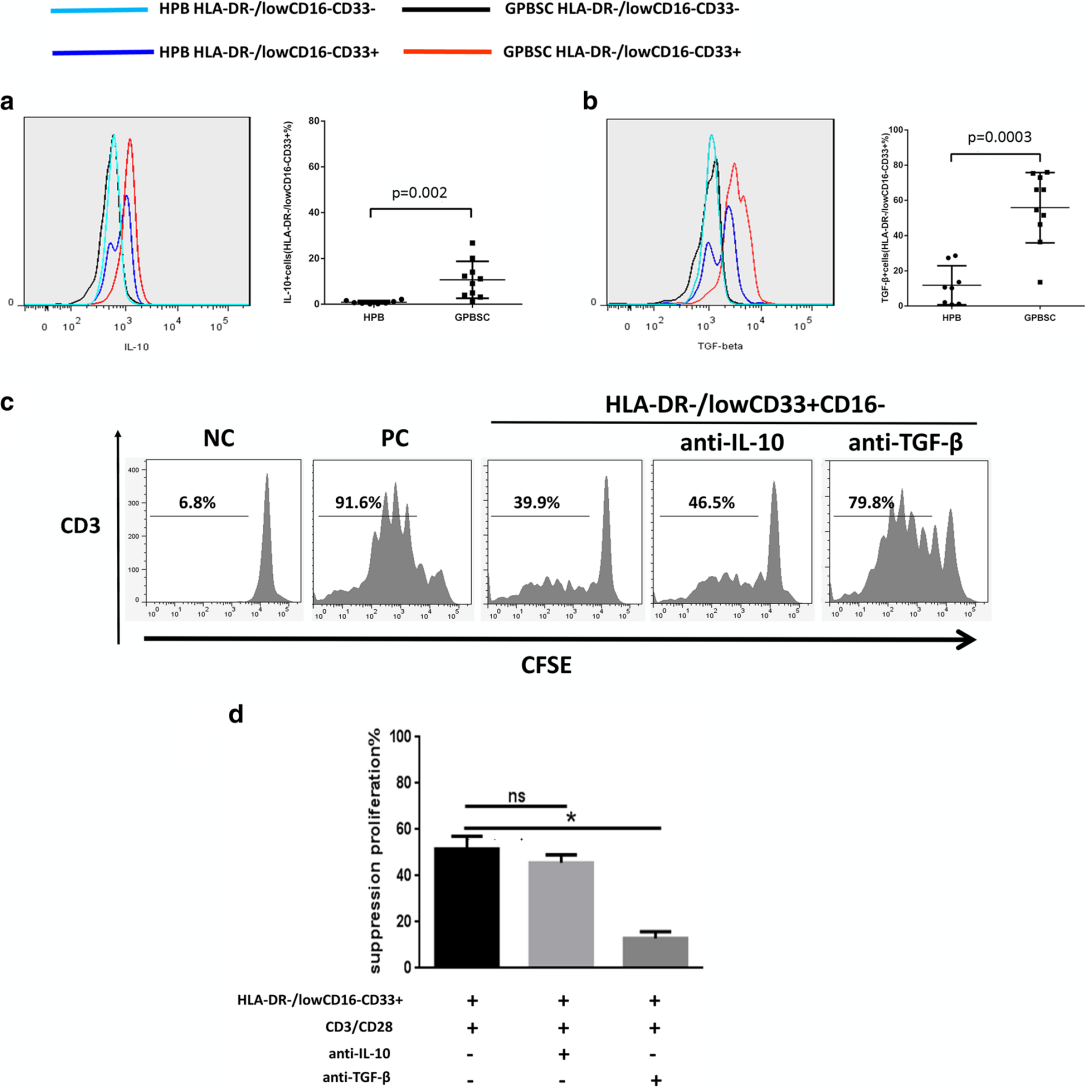
The percentage of IL-10^+^ and TGF-β^+^ cells increased in HLA-DR^−/low^CD33^+^CD16^−^ cells in GPBSC and HLA-DR^−/low^CD33^+^CD16^−^ cells suppressed T cell proliferation in a TGF-β-dependent manner. **a**, **b** IL-10 and TGF-β were analyzed by cytometry in HPB and GPBSC from eight healthy donors. IL-10^+^ and TGF-β^+^ cells were gated on HLA-DR^−/low^CD33^+^CD16^−^cells. **c** HLA-DR^−/low^CD33^+^CD16^−^ cells sorted from GPBSC and co-cultured with autologous CD3^+^ T cells, with or without anti-IL-10 antibody or anti-TGF-β antibody in culture system in the presence of anti-CD3/CD28 beads. The ratio of HLA-DR^−/low^CD33^+^CD16^−^ to CD3^+^ T cells was 1:1. Co-cultured for 4 days, T cell proliferation was evaluated using CFSE labeling. Unstimulated T cells were used as a negative control. Stimulated T cells were used as a positive control. **d** The percentage of T cell suppression was shown in a different group. Data were compared using Student’s unpaired *t* test (ns, not significant; *p ≤ 0.05). **d** Percentages of T cell suppression in different groups. Data were compared using Student’s unpaired *t* test (ns, not significant; *p ≤ 0.05)

To investigate the mechanisms underlying the inhibition of T cell proliferation, the IL-10- and TGF-β-suppressive cytokines expressed by G-CSF-mobilized HLA-DR^−/low^CD33^+^ CD16^−^ cells were detected. The CD33^−^ fraction and non-mobilized HLA-DR^−/low^CD33^+^CD16^−^ cells served as controls. The median percentage of IL-10 or TGF-β positive cells among HLA-DR^−/low^CD33^+^CD16^−^ population in the G-PBSC was significantly higher than those of H-PB (IL-10, 10.1% vs. 0.62%, *p* = 0.002; TGF-β, 60.24% vs. 10.39%, *p* = 0.0003). Notably, the TGF-β^+^ cells among the HLA-DR^−/low^CD33^+^CD16^−^ cells significantly increased compared to the CD33^−^ fraction or non-mobilized HLA-DR^−/low^CD33^+^CD16^−^ cells (Fig. 2a, b).

Subsequently, the inhibition of CD3^+^ T cell proliferation by HLA-DR^−/low^CD33^+^CD16^−^ cells was markedly reduced in the presence of an anti-TGF-β antibody but not an anti-IL-10 antibody, indicating that the immunosuppressive activity relied more on the presence of TGF-β (Fig. 2c, d).

Other immunosuppressive mechanisms (arginase, iNOS, IDO, and COX2) were excluded by adding selective inhibitors of arginase (nor-NOHA (*N*(w)-hydroxy-nor-l-arginine)), iNOS (L-NMMA), IDO (NLG8189) to the co-cultures. However, no reverse effect on T cell proliferation was observed (Additional file 1: Figure S3).

### HLA-DR^−/low^CD33^+^CD16^−^ cells modulate Th cell balance and induce Treg generation

**Fig. 3:**
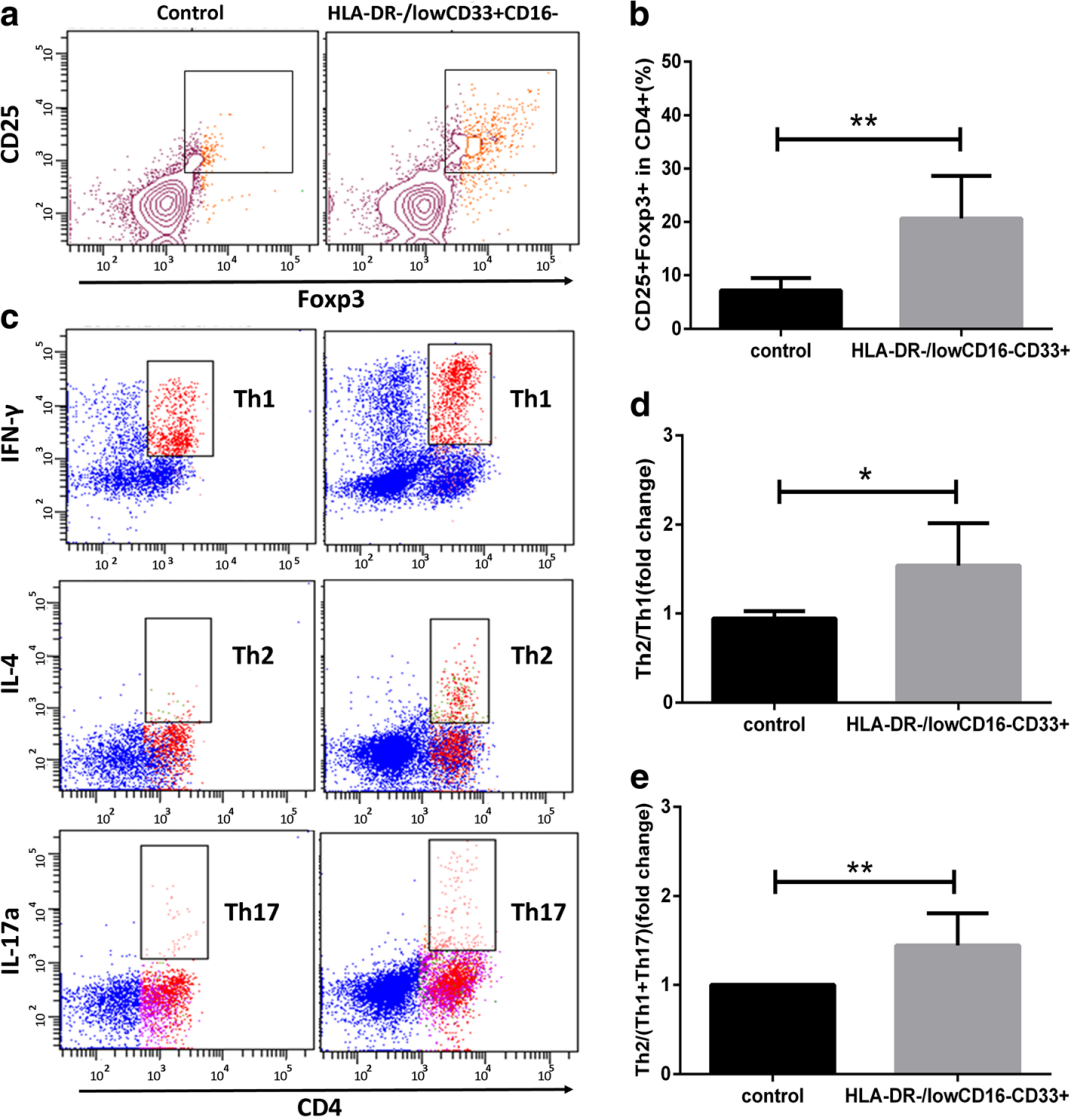
HLA-DR^−/low^CD33^+^CD16^−^ cells could affect the balance of Th subsets induce Treg generation*.* **a** Treg (CD4^+^CD25^+^Foxp3^+^) generation was detected through flow cytometry. CD3^+^ T cells were co-cultured with or without HLA-DR^−/low^CD33^+^ CD16^−^ cells for 4 days. **b** The percentage of Treg in CD4^+^ T cell, group HLA-DR^−/low^CD33^+^CD16^−^ vs. control, *p* = 0.0048. **c** Flow cytometry analysis of Th1 (CD4^+^IFN-γ^+^), Th2 (CD4^+^IL-4^+^), Th17 (CD4^+^IL-17a^+^). CD3^+^ T cells were co-cultured with or without HLA-DR^−/low^CD33^+^CD16^−^ cells sorted from GPBSC in the presence of CD3/CD28 for 4 days. Red for CD4+ cell fraction and blue for CD4-negative part. **d** Th2/Th1 ratio fold change compared with the control group, *p* = 0.0159. **e** Th2/(Th1+Th17) ratio fold change compared with the control group, *p* = 0.0095

The present study demonstrated that CD4^+^CD25^+^Foxp3^+^Treg cells increased in the HLA-DR^−/low^CD33^+^CD16^−^ cell co-culture group (Fig. 3a, c). Th cell subsets were analyzed after co-culture with or without HLA-DR^−/low^CD33^+^CD16^−^ cells. Though the expression of IFN-γ and IL-17A increased, co-culture with HLA-DR^−/low^CD33^+^CD16^−^ cells led to a significantly higher ratio of Th2/Th1 and Th2/Th1+Th17 cells than the control group (Fig. 3b, d, e).

### HLA-DR^−/low^CD33^+^CD16^−^ MDSCs prevent aGVHD in humanized mouse model

**Fig. 4:**
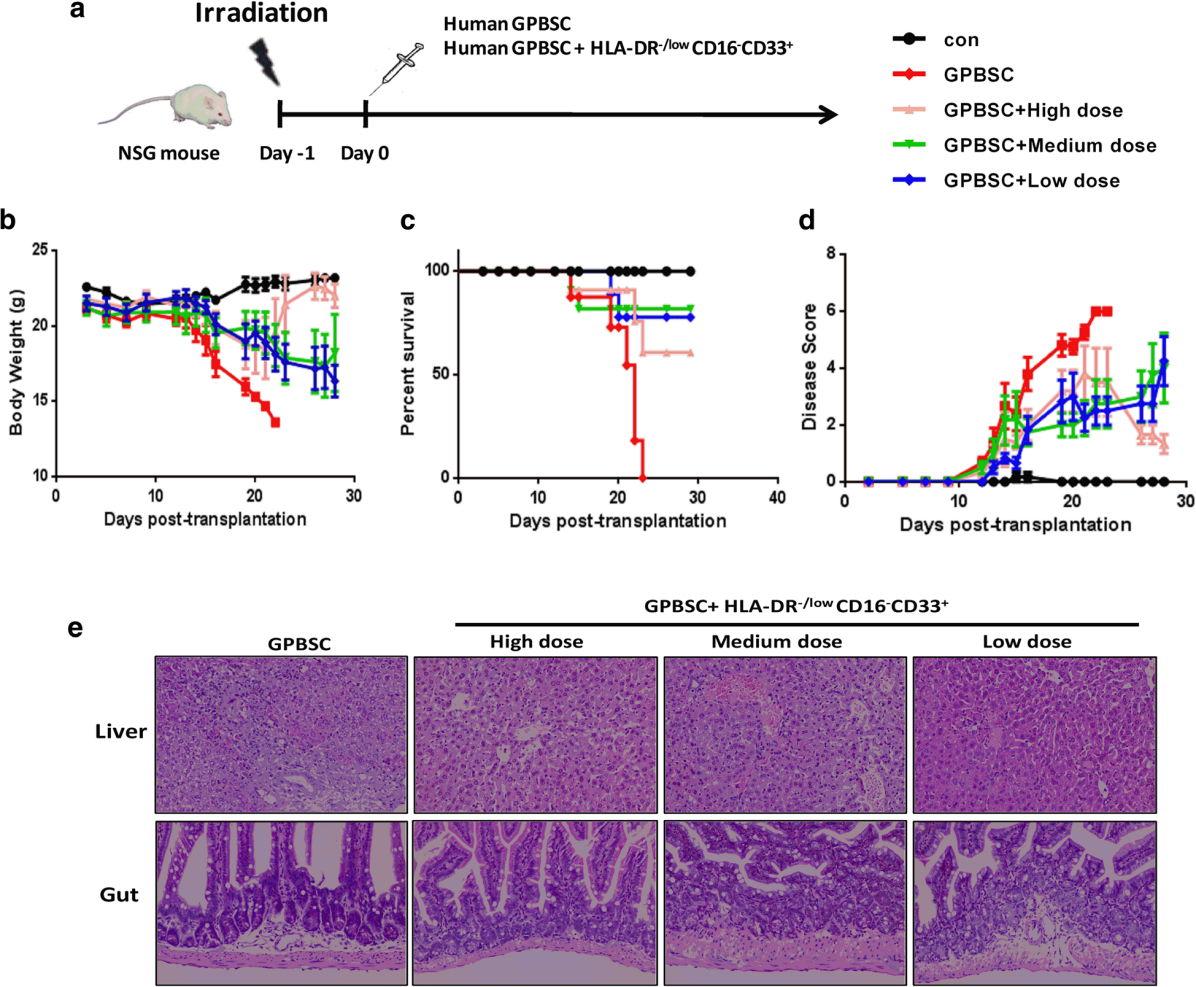
HLA-DR^−/low^CD33^+^CD16^−^ cells could inhibit the development of aGVHD in humanized mouse model. **a** Protocol for the establishment of aGVHD model through injecting human GPBSC. Sublethally irradiated NSG mice received 5 × 10^6^ human GPBSC with or without 2.5 × 10^6^, 1 × 10^6^, and 5 × 10^5^ G-CSF-mobilized HLA-DR^−/low^CD33^+^CD16^−^ sorted from GPBSC. **b** Weight Loss, **c** median survival, and **d** pathological score in xenografted mice were shown. Mice co-transplanted with HLA-DR^−/low^CD33^+^CD16^−^ (pink, green, and blue, eight mice each group) had improved survival after transplant as compared with GPBSC-injected group (red). For survival, weight change, and disease score: GPBSC + high dose versus GPBSC, GPBSC + medium dose versus GPBSC, and GPBSC + low dose versus GPBSC, *p* < 0.05. **e** Representative histology of the target organs harvested on day 22 after transplantation. All results were derived from two pooled independent experiments. Each experiment was performed with G-PBSC from one donor for all recipients. Results were presented and compared with Kaplan-Meier survival curves

**Fig. 5:**
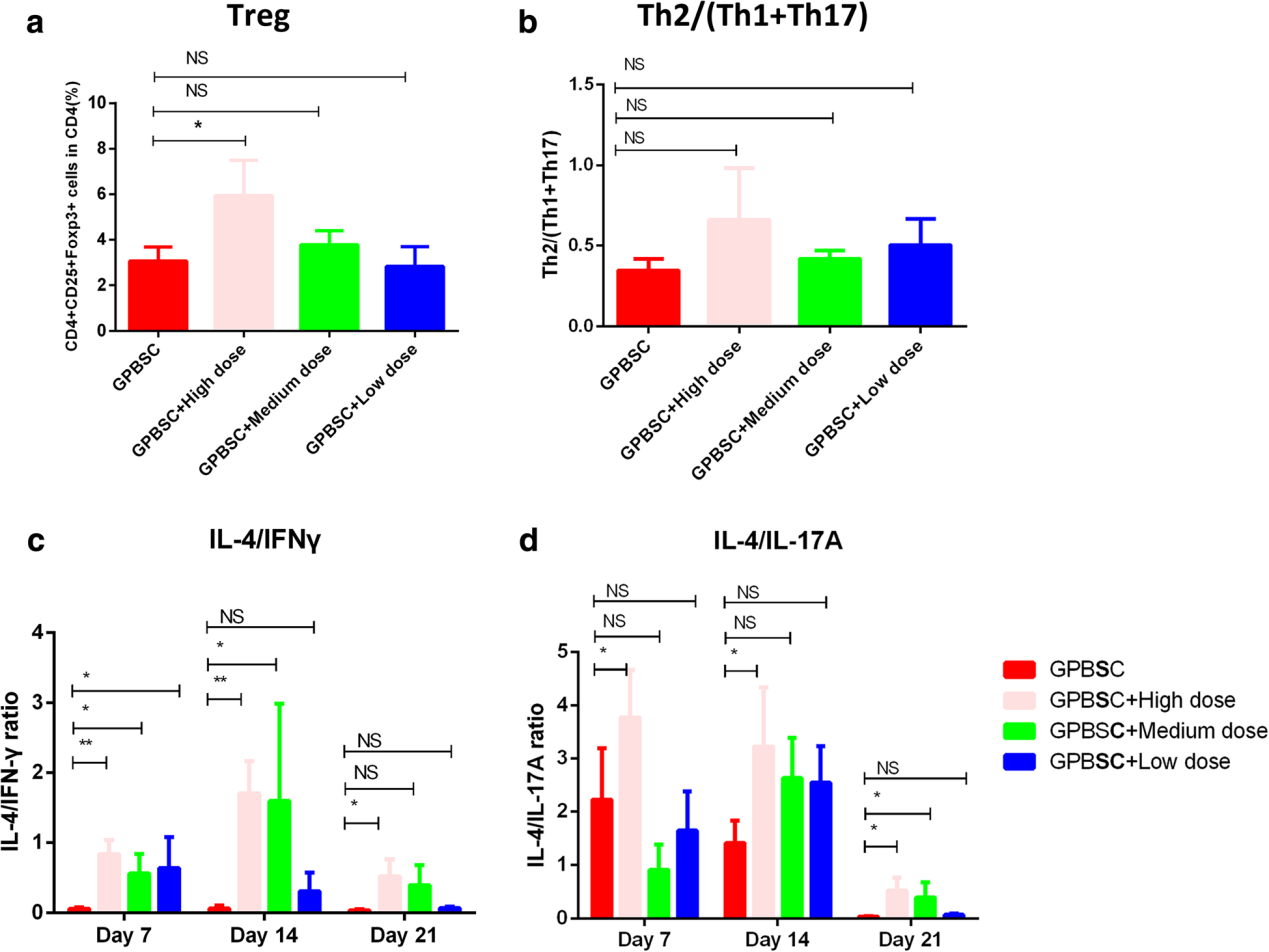
T cell subsets and cytokines detected in the peripheral blood in NSG mice at day 21. **a** Percentage of Treg in CD4 cells between intragroup. **b** Th2/(Th2+Th17) ratios between intragroup. **c** IL-4/INF-γ ratios between intragroup. **d** IL-4/IL-17A ratios between intragroup. *p < 0.05; **p < 0.01

Because G-CSF expanded HLA-DR^−/low^CD33^+^CD16^−^ cells into the peripheral blood and exerted a strong regulatory effect on T cell immune response, we next investigated if such population prevents aGVHD in vivo. To mimic aGVHD after allo-HSCT in humans, a humanized xenogeneic aGVHD model was established by injection of 5 × 10^6^ donor G-PBSC into irradiated non-obese diabetic (NOD)-severe combined immune deficient (SCID)-IL-2Rg−/− mice (NSG mice) [38, 39] (Fig. 4a). Human white blood cell engraftment was detected by CD45^+^ cells at 7, 14, and 21 days after transplantation, and there was no significant difference of human cells engraftment intragroup (14 days, *p* = 0.486; 21 days, *p* = 0.4012) (Additional file 1: Figure S4). We failed to detect the engraftment of HLA-DR^−/low^CD33^+^CD16^−^ MDSCs in PB as these cells could be detected in the spleen and bone marrow at 14 days after transplantation; however, they decreased quickly and almost could not be detected at 21 days after co-transplantation (Additional file 1: Figure S5). Acute GVHD occurred in the humanized mice receiving donor G-PBSC during 2 to 4 weeks after transplantation as evidenced by weight loss, disease score (hunching, activity, ruffling, and diarrhea), and death. Similar to humans [40, 41], humanized mice with aGVHD showed severe inflammation, leukocyte infiltration, necrosis, and tissue damage in target organs, such as the skin, liver, and gut in the G-PBSC group (Fig. 4e). On the base of this model, a prevention model was established by one-time co-infusion of 2.5 × 10^6^, 1 × 10^6^, and 5 × 10^5^ human G-CSF-mobilized HLA-DR^−/low^CD33^+^CD16^−^cells sorted from the same donor with graft at the same day. All of these cell doses reduced weight loss (Fig. 4b), improved overall survival (Fig. 4c), improved pathological score (Fig. 4d), and lessened tissue damage in target organs in medium and high doses of HLA-DR^−/low^CD33^+^ CD16^−^cells compared to mice grafted without or with low-dose human HLA-DR^−/low^CD33^+^ CD16^−^ cells (Fig. 4e). For human Treg detected at 21 days after transplantation, a significant difference was found between the high-dose HLA-DR^−/low^CD33^+^CD16^−^ cells and GPBSC control (*p* = 0.0498); there was no disparity between medium dose vs. control (*p* = 0.6825) and low dose vs. control (*p* > 0.9) (Fig. 4a, Additional file 1: Figure S6); there were no disparity of Th2/(Th1+Th17) ratio between the different groups (Fig. 4b, Additional file 1: Figure S6). For cytokines detected at 7, 14, and 21 days after transplantation, there were significantly less INF-γ and IL-6 at days 7, 14, and 21 and IL-17A and TNF-α at days 14 and 21 (high dose of HLA-DR^−/low^CD33^+^CD16^−^ cells vs. control) and less INF-γ and TNF-α at days 7 and 21, IL-17A at days 7 and 14, and IL-6 at day 21 (medium dose of HLA-DR^−/low^CD33^+^CD16^−^ cells vs. control) (Additional file 1: Figure S7). IL-4/INF-γ and IL-4/IL-17A ratios were higher in the high-dose HLA-DR^−/low^CD33^+^CD16^−^ cells group vs. control at 7, 14, and 21 days after transplantation; by comparison with medium-dose HLA-DR^−/low^CD33^+^ CD16^−^ cells group vs. control, IL-4/INF-γ ratios were higher at days 7 and 14, while IL-4/IL-17A ratio was higher at day 21 (Fig. 5c, d). These results confirmed that immune-suppressive activity of HLA-DR^−/low^CD33^+^CD16^−^ cells which might contribute to alleviating aGVHD in NSG model.

### Negative correlations between the frequency of HLA-DR^−/low^CD33^+^CD16^−^ cells among donor PBSCs and the development of aGVHD in recipients

In a prospective study (registered at www.chictr.org as ChiCTR-OCH-10000940), 100 allo-HSCT recipients were enrolled to assess the role of HLA-DR^−/low^CD33^+^CD16^−^ in graft on the occurrence of aGVHD. Of these patients, 76 patients received a combination of G-BM and G-PBSC grafts from haplo-donors, and 24 patients received grafts from MSDs. The indications for allo-HSCT were as follows: AML (*n* = 39), ALL (*n* = 29), MDS (*n* = 8), SAA (*n* = 14), and lymphoma (*n* = 10). Ninety-nine (99%) patients reached full donor chimerism before day 30 after transplantation as assessed by the analysis of a variable number of tandem repeats on the peripheral blood. Platelet recovery (count > 20,000/ml) was achieved at a median of day 32 post-transplantation, whereas neutrophils reached values greater than 500/ml on day 12. Only 1 patient died due to transplant-related mortality at day 11 post-HSCT. The median follow-up period of the enrolled patients was 11 months. The cell components in the graft were listed in Additional file 1: Table S1. Patients were divided into 2 groups according to the median absolute counts of HLA-DR^−/low^CD33^+^CD16^−^ MDSCs in grafts (1.88 × 10^7^/kg) as follows: 53 patients were in the high MDSCs group (≥ 1.88 × 10^7^/kg), and 47 patients were in the low MDSCs group (Table 2).

**Table 2:** Patient, donor, and transplant characteristics

	MDSCs low	MDSCs high	*p*
Number	47	53	
Patient age, median (range)	32 (5–62)	30 (2–53)	NS
Patient sex, male (%)	30 (63.8%)	29 (54.7%)	NS
Diagnosis			NS
AML, *n* (%)	21 (44.7%)	18 (34.0%)	
ALL, *n* (%)	13 (27.7%)	16 (30.2%)	
MDS, *n* (%)	3 (6.4%)	5 (9.4%)	
SAA, *n* (%)	6 (12.8%)	8 (15.1%)	
Lymphoma or myeloma, *n* (%)	4 (8.5%)	6 (11.3%)	
Disease Risk Index (DRI) overall			NS
Low, *n* (%)	3 (6.7%)	2 (4.4%)	
Intermediate, *n* (%)	5 (12.2%)	7 (15.5%)	
High, *n* (%)	29 (70.7%)	32 (63.4%)	
Very high, *n* (%)	4 (9.8%)	4 (8.9%)	
Donor Type			
MSD, *n* (%)	13 (27.7%)	11 (20.8%)	NS
Haplo, *n* (%)	34 (72.3%)	42 (79.2%)	NS
1 Locus, *n* (%)	1 (2.1%)	0 (0%)	
2 Locus, *n* (%)	2 (4.3%)	3 (5.7%)	
3 Locus, *n* (%)	31 (65.9%)	39 (73.6%)	
Engraftment			
WBC + days, median (range)	14 (10–22)	12 (10–24)	
PLT + days, median (range)	14 (7–32)	13.5 (8–63)	
Cells in allograft			
CD34^+^ (× 10^6^/kg), median (range)	1.92 (0.62–5.85)	3.14 (0.64–6.85)	0.002
CD3^+^ T (× 10^8^/kg), median (range)	2.31 (0.61–3.79)	2.63 (0.82–5.79)	NS
CD4^+^ (× 10^8^/kg), median (range)	1.21 (0.32–2.12)	1.49 (0.43–3.42)	NS
CD8^+^ (× 10^8^/kg), median (range)	0.72 (0.15–1.87)	0.92 (0.31–2.29)	NS

*AML* acute myeloid leukemia, *ALL* acute lymphoid leukemia, *MDS* myelodysplastic syndromes, *SAA* severe aplastic anemia, *NS* not significant

**Fig. 6:**
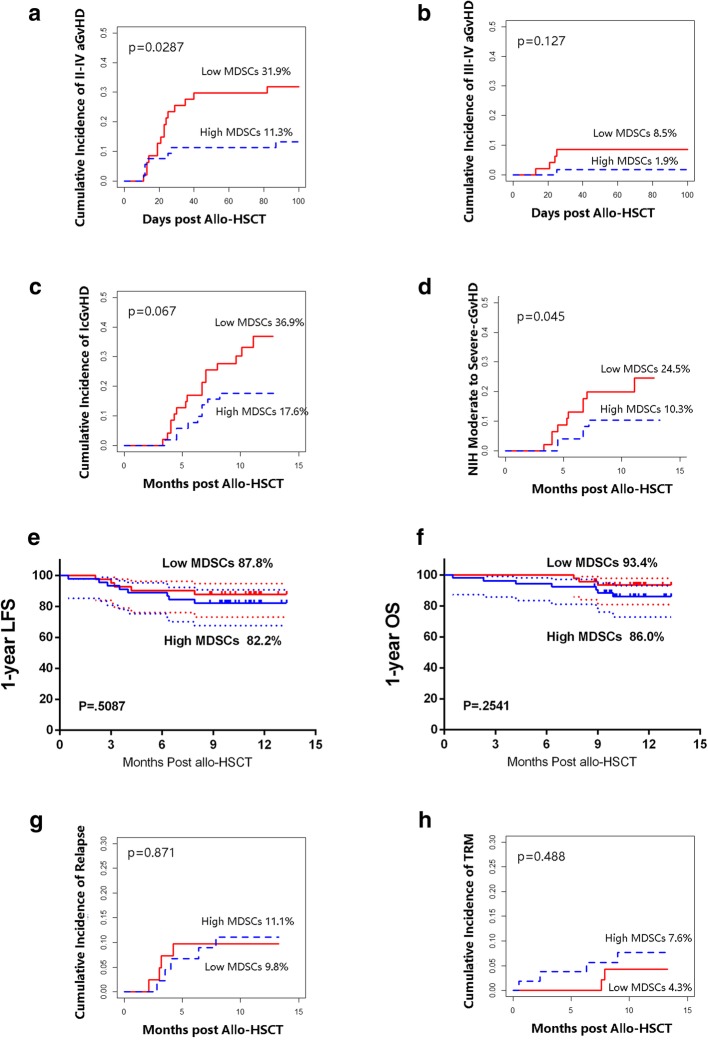
Association of HLA-DR^−/low^CD33^+^CD16^−^ cells and clinical outcomes. The cumulative incidences of aGvHD for patients were calculated according to competitive risk. Gray’s test was used in the cumulative incidence analyses. The “low” and “high” groups were separated according to the median of HLA-DR^−/low^CD33^+^CD16^−^ MDSC absolute numbers in the graft (< vs. ≥ 1.88 × 10^7^/kg). **a** II–IV aGvHD in total allo-HSCT cohort (*n* = 100). **b** III-IV aGVHD in allo-HSCT cohort. **c** cGvHD in allo-HSCT cohort. **d** NIH moderate to severe cGvHD in allo-HSCT cohort. **e** LFS in hematological malignancies. **f** OS in allo-HSCT cohort. **g** CIR in hematological malignancies. **h** TRM in allo-HSCT cohort

The cumulative incidences for different grades of aGVHD at 100 days after transplantation for the total cohort were as follows: 50% of patients developed grade I–IV aGVHD; 28% of patients developed grade I aGVHD (61.8% for haplo-HSCT and 12.5% for MSD-HSCT); 17% of patients had grade II aGVHD (25% for haplo-HSCT and 12.5% for MSD-HSCT); and 5% of patients developed grade III–IV aGVHD (5.3% for haplo-HSCT and 4.2% for MSD-HSCT). Patients who received a high number of MDSCs exhibited lower incidence of grade II–IV aGVHD compared to the low MDSC groups in allo-HSCT (11.3% vs. 31.9%, *p* = 0.0287) and comparable of grade III–IV aGVHD in allo-HSCT (1.9% vs. 8.5%, *p* = 0.127) (Fig. 6a, b). In the bivariable analysis, high MDSC dose and CD34^+^ cells in the graft were interacted; for consideration of collinearity in multiple variable analysis (MVA), backward elimination process was applied to choose one factor (high MDSC dose) which was taken into the final MVA model. In the multivariate analysis, absolute counts of MDSCs in allografts emerged as the only independent factor that reduced the occurrence of grades II–IV (HR 0.388, 95% CI 0.158–0.954, *p* = 0.039). Age, patient gender, HLA disparity, ABO disparity, patient-donor relationship, and CD3/CD4/CD8/CD14/CD34 cells in grafts were not correlated to grades II–IV in the analysis.

For patients with leukemia-free survival (LFS) above 100 days post-transplantation, the estimated 1-year cumulative incidences for chronic GVHD (cGVHD) of the total cohort were as follows: 25.5% patients developed cGVHD, 8.2% patients with extensive cGVHD, and 15.3% patients with moderate to severe cGvHD with NIH criteria. There were trends that patients who received a high number of MDSCs exhibited a lower incidence of cGVHD (17.6% vs. 36.9%, *p* = 0.067) and NIH moderate to severe cGvHD (10.3% vs. 24.5%, *p* = 0.045) (Fig. 6c, d). In the univariate analysis, high- vs. low-dose MDSCs are identified as a potential factor that reduced the occurrence of cGVHD (HR 0.481, 95% CI 0.213–1.089, *p* = 0.072). In the multivariate analysis, no independent risk factor was found.

The estimated 1-year LFS (82.2% vs. 87.8%, *p* = 0.508) and 1-year cumulative incidence of relapse (CIR, 11.1% vs. 9.8%, *p* = 0.871) were comparable in patients with hematological malignancies (*n* = 86) who received high or low dose of MDSCs (Fig. 6e, g). The estimated 1-year overall survival (OS, 86.0% vs. 93.4%, *p* = 0.254) and 1-year treatment-related mortality (TRM, 7.6% vs. 4.3%, *p* = 0.488) were comparable in all allo-HSCT recipients (*n* = 100) with high or low dose of MDSCs (Fig. 6f, h). In the multivariate analysis, DRI is the only potential independent risk factor identified for CIR (HR 2.788, HR 0.905–5.892, *p* = 0.044); no independent factor was found for LFS, OS, and TRM.

## Discussion

**Fig. 7:**
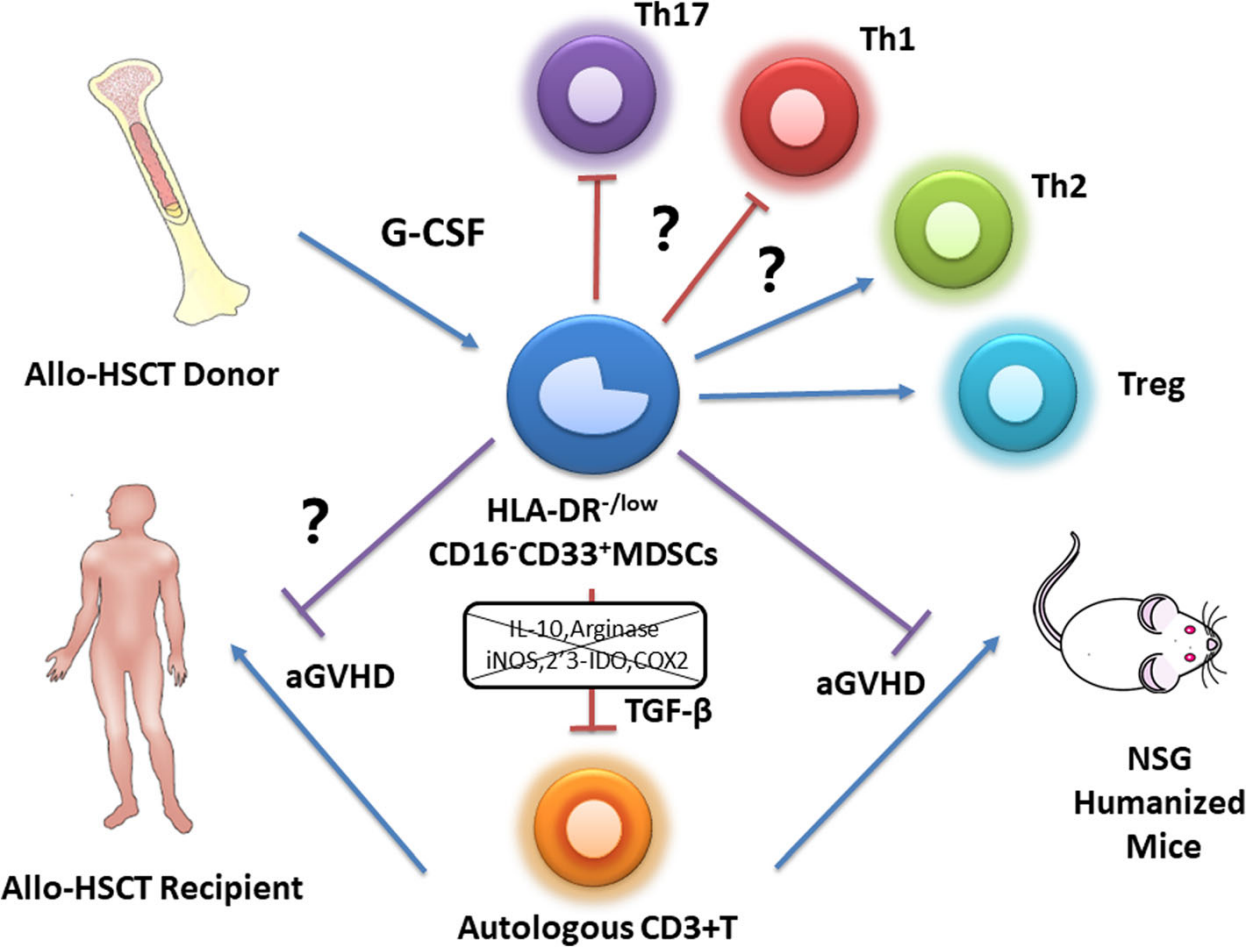
Summary of study. Early myeloid-derived suppressor cells (HLA-DR^−/low^CD33^+^CD16^−^) expanded by granulocyte colony-stimulating factor derived from G-CSF-mobilized donor PBSCs. This MDSC population suppressed T cell proliferation in vitro and in humanized mouse model, potentially by TGF-β-dependent manner, and promoted Treg generation, which also prevents acute graft-versus-host disease (GVHD) in humanized mouse and might contribute to lower GVHD in patients post-allo-HSCT

The present study characterized a previously unidentified immature monocytic cell population defined by the cell surface expression of HLA-DR^−/low^CD33^+^CD16^−^ in healthy donors after G-CSF treatment. These cells represented an eMDSC population. Compared with the same compartment in non-mobilized PB, the percentage of IL-10^+^ cells and TGF-β^+^ cells significantly increased, suggesting that G-CSF not only expanded these cells but also endowed them immunosuppressive properties and regulatory function dependent on TGF-β. Importantly, these cells prevented aGVHD in a humanized mouse model. Moreover, the prospective cohort suggested that these eMDSCs in the donor graft inversely correlated with the incidence and severity of aGVHD in recipients. These cells may be a potential treatment for immunomodulatory prevention and therapy for aGVHD in allo-HSCT (Fig. 7).

MDSCs represent a heterogenic population of immature myeloid cells, and the definition of these subsets has not yet reached consensus [42–44]. Currently, most MDSC phenotypes have been reported to be generated in the tumor state [29, 45]. There are fewer reports about the generation and function of MDSCs in regions other than tumors and chronic inflammation. GM-CSF is considered as a major growth factor driving myelopoiesis [46, 47]. In combination with IL-6, IL-1β, prostaglandin (PG) E2, TNF-α, or VEGF, GM-CSF has been reported to mediate the generation of highly suppressive MDSCs from CD33^+^ peripheral blood mononuclear cells isolated from healthy donors [48]. Importantly, GM-CSF and IL-6 allow a rapid and efficient generation of MDSCs with strong tolerogenic activity from the precursors present in mouse and human bone marrow [49]. Solito demonstrated that the GM-CSF, G-CSF, and IL-6 cytokines allow a rapid generation of MDSCs from precursors present in human BM in vitro and that the CD11b^low/−^/CD16^−^-immature subset is the only subset responsible for immune suppression in BM-MDSCs [29]. As G-CSF is more widely applied in an allo-HSCT setting, Luyckx et al. reported that expanding myeloid cells in the peripheral blood of G-CSF-mobilized donors have the typical more-differentiated phenotype of the mononuclear and G-MDSC subtypes, and they confirmed their suppressive properties [24]. Vendramin et al. demonstrated that G-CSF-expanded myeloid cells display the phenotype of M-MDSCs and suppress alloreactive T cells [25]. However, these MDSCs have not been tested in an in vivo model. In contrast, the present study demonstrated that HLA-DR^−/low^CD33^+^CD16^−^ eMDSCs prevented acute GVHD in a humanized mouse model in vivo. As G-CSF treatment alone is significantly associated with the generation of HLA-DR^−/low^CD33^+^CD16^−^ eMDSCs in both frequency and absolute number compared to only increasing number in total MDSCs (Lin^−^HLA-DR^−/low^CD11b^+^CD33^+^), M-MDSCs (HLA-DR^-low^CD11b^+^CD33^+^CD14^+^CD15^−^), and G-MDSCs (HLA-DR^−/low^CD11b^+^CD33^+^CD14^−^CD15^+^), we speculated that G-CSF-expanded HLA-DR^−/low^CD33^+^ CD16^−^ eMDSCs accumulated in G-PBSC may play an important role in the G-CSF-induced immune tolerance in allo-HSCT.

Immune-suppressive activity contributes to the definition of MDSCs; though the activity of HLA-DR^−/low^CD33^+^CD16^−^ eMDSCs was lower compared with M/G-MDSCs, it suggested the underlining mechanism of the immunosuppressive effects of HLA-DR^−/low^CD33^+^CD16^−^ eMDSCs on T cells in vitro might be distinct from M/G-MDSCs [24, 25]. Mougiakakos suggested that indoleamine 2,3-dioxygenase (IDO) plays a critical role in the immunosuppressive effects of CD14^+^HLA-DR^low/neg^ M-MDSCs [28]. In contrast, the present study showed that the inhibition of CD3^+^ T cell proliferation by HLA-DR^−/low^CD33^+^CD16^−^ eMDSCs was abolished in the presence of an anti-TGF-β antibody rather than an anti-IL-10 antibody. This result should be interpreted carefully as there were 10% IL-10^+^ while 60% TGF-β^+^ HLA-DR^−/low^CD33^+^CD16^−^ cells in G-PBSC. These results implied that the immunosuppressive activity of HLA-DR^−/low^CD33^+^CD16^−^ eMDSCs might depend more on TGF-β, which may further strengthen this new set of eMDSCs compared to traditional MDSCs.

Acute GVHD is mediated by donor-derived T cells and activation with alloantigens expressed on host tissues. Donor-derived naive CD4^+^ T cells differentiate into Th cell subsets of effector T cells, such as Th1 and Th17, expressing distinct sets of transcriptional factors and cytokines, which mediate organ-specific GVHD. Previous studies have demonstrated that Th2 cells alleviate aGVHD in mouse models [50, 51]. While playing key roles in suppressing autoimmunity and maintaining immune homeostasis, Treg reduce the severity of GVHD. The effects of Treg on inhibiting aGVHD have been reported in several previous studies [52–54]. The present study suggested that the Treg induction and polarization of T cells from Th1/Th17 to Th2 should be primarily regulated by the HLA-DR^−/low^CD33^+^CD16^−^ eMDSCs. Although, the inverse ratio of Th2/(Th1+Th17) might be interpreted with caution as there was an increase in all cytokines measured including IFN-γ and IL-17 in vitro; there were significantly less INF-γ, IL-17A, IL-6, and TNF-α at days 14 and 21 in NSG mice after infusion of high-dose HLA-DR^−/low^CD33^+^CD16^−^ eMDSCs, with increased IL-4/INF-γ and IL-4/IL-17 ratio. In the present study, HLA-DR^−/low^CD33^+^CD16^−^ eMDSCs were demonstrated to prevent aGVHD by evaluating weight loss, overall survival, pathological score, and less tissue damage of the gut and liver; it should be noticed that the lung and bone marrow are also the main target organs of GVHD in NSG model and might be evaluated in the future [55]. In summary, it is anticipated that donor-derived HLA-DR^−/low^CD33^+^CD16^−^ eMDSCs may prevent aGVHD via TGF-β activity, inducing Treg generation, and possibly by modulating Th cell balance.

In addition, several studies have reported that MDSCs modulate the function of alloreactive T cells and prevent GVHD without impairing the graft-versus-leukemia (GVL) effects in murine models [15, 56]. In our prospective cohort study, high dose of HLA-DR^−/low^CD33^+^CD16^−^ eMDSCs was identified as independent risk factor for II–IV aGVHD, while it is also associated with lower risk of NIH moderate to severe cGVHD while did not affect CIR and LFS. As the high MDSC group also had significantly more CD34^+^ cells in the graft, though backward elimination process had been applied to reduce collinearity, high CD34^+^ cells might promote platelet engraftment and lymphocyte recovery after allo-HSCT and have mixed influence on the association of MDSCs with clinical outcome. In addition, as the median follow-up is still relatively short, it is also important to further understand if the donor-derived cells in the present study can prevent cGVHD without impairing the GVL effects in the future.

In conclusion, the present study identified and confirmed a population of previously unreported immature myeloid regulatory cells derived from G-CSF-mobilized donor PBSCs. This MDSC-like population suppressed T cell proliferation in a TGF-β-dependent manner, modulated the T helper cell balance from Th1 to Th2, and promoted Treg generation. These effects are beneficial for establishing immune tolerance in HSCT and may prevent aGVHD. Therefore, the mechanisms underlying the prevention of aGVHD in recipients and the immunomodulatory function of these donor-derived cells need to be studied in the future. Such work might improve the transplantation outcomes with allo-HSCT and facilitate the potential use of these cells in the clinic.

## Additional file

Additional file 1: **Table S1.** Cell components in graft. **Figure S1.** HLA-DR^−/low^CD33^+^CD16^−^ eMDSCs demonstrated superior immune-suppressive activity compared with CD33^−^ and HLA-DR^−/low^CD33^+^CD16^+^ fraction. **Figure S2.** Comparison of immune-suppressive activity of HLA-DR^−/low^CD33^+^CD16^−^ eMDSCs with M-MDSCs and G-MDSCs. **Figure S3.** Selective inhibitors of arginase (nor-NOHA), iNOS (L-NMMA), and IDO (NLG8189) on T cell proliferation. **Figure S4.** Human white blood cell engraftment at 7 days, 14 days, and 21 days after co-transplantation. **Figure S5.** eMDSC engraftment in NSG mice at 14 and 21 days after co-transplantation. **Figure S6.** Treg, Th1, Th2, and Th17 cells detected in the peripheral blood in NSG mice at day 21. **Figure S7.** Cytokines detected in the peripheral blood of NSG mice at 7, 14, and 21 days after co-transplantation. (DOCX 724 kb)

## Abbreviations

aGVHD: Acute graft-versus-host disease
ALL: Acute lymphoid leukemia
allo-HSCT: Allogeneic hematopoietic stem cell transplantation
AML: Acute myeloid leukemia
DRI: Disease Risk Index
G-CSF: Granulocyte colony-stimulating factor
GRFS: GVHD-relapse-free survival
MDS: Myelodysplastic syndromes
MDSCs: Myeloid-derived suppressor cells
MSDs: Matched sibling donors
NSG: Non-obese diabetic (NOD)-severe combined immune deficient (SCID)-IL-2Rg−/−
SAA: Severe aplastic anemia
TGF-β: Transforming growth factor-beta

## References

[1] Gratwohl A, Baldomero H, Aljurf M, Pasquini MC, Bouzas LF, Yoshimi A, Szer J, Lipton J, Schwendener A, Gratwohl M (2010). Hematopoietic stem cell transplantation: a global perspective. JAMA.

[2] Zeiser R, Blazar BR (2017). Acute graft-versus-host disease - biologic process, prevention, and therapy. N Engl J Med.

[3] Blazar BR, MacDonald KPA, Hill GR (2018). Immune regulatory cell infusion for graft-versus-host disease prevention and therapy. Blood.

[4] Marin-Acevedo JA, Soyano AE, Dholaria B, Knutson KL, Lou Y (2018). Cancer immunotherapy beyond immune checkpoint inhibitors. J Hematol Oncol.

[5] Lai P, Chen X, Guo L, Wang Y, Liu X, Liu Y, Zhou T, Huang T, Geng S, Luo C (2018). A potent immunomodulatory role of exosomes derived from mesenchymal stromal cells in preventing cGVHD. J Hematol Oncol.

[6] Condamine T, Gabrilovich DI (2011). Molecular mechanisms regulating myeloid-derived suppressor cell differentiation and function. Trends Immunol.

[7] Wang D, Yu Y, Haarberg K, Fu J, Kaosaard K, Nagaraj S, Anasetti C, Gabrilovich D, Yu XZ (2013). Dynamic change and impact of myeloid-derived suppressor cells in allogeneic bone marrow transplantation in mice. Biol Blood Marrow Transplant.

[8] Bronte V, Brandau S, Chen SH, Colombo MP, Frey AB, Greten TF, Mandruzzato S, Murray PJ, Ochoa A, Ostrand-Rosenberg S (2016). Recommendations for myeloid-derived suppressor cell nomenclature and characterization standards. Nat Commun.

[9] Fang P, Li X, Dai J, Cole L, Camacho JA, Zhang Y, Ji Y, Wang J, Yang XF, Wang H (2018). Immune cell subset differentiation and tissue inflammation. J Hematol Oncol.

[10] Talmadge JE, Gabrilovich DI (2013). History of myeloid-derived suppressor cells. Nat Rev Cancer.

[11] Ochoa AC, Zea AH, Hernandez C, Rodriguez PC (2007). Arginase, prostaglandins, and myeloid-derived suppressor cells in renal cell carcinoma. Clin Cancer Res.

[12] Almand B, Clark JI, Nikitina E, van Beynen J, English NR, Knight SC, Carbone DP, Gabrilovich DI (2001). Increased production of immature myeloid cells in cancer patients: a mechanism of immunosuppression in cancer. J Immunol.

[13] Rutella S, Zavala F, Danese S, Kared H, Leone G (2005). Granulocyte colony-stimulating factor: a novel mediator of T cell tolerance. J Immunol.

[14] Jun HX, Jun CY, Yu ZX (2004). In vivo induction of T-cell hyporesponsiveness and alteration of immunological cells of bone marrow grafts using granulocyte colony-stimulating factor. Haematologica.

[15] MacDonald KP, Rowe V, Clouston AD, Welply JK, Kuns RD, Ferrara JL, Thomas R, Hill GR (2005). Cytokine expanded myeloid precursors function as regulatory antigen-presenting cells and promote tolerance through IL-10-producing regulatory T cells. J Immunol.

[16] Pan L, Delmonte J, Jalonen CK, Ferrara JL (1995). Pretreatment of donor mice with granulocyte colony-stimulating factor polarizes donor T lymphocytes toward type-2 cytokine production and reduces severity of experimental graft-versus-host disease. Blood.

[17] Franzke A, Piao W, Lauber J, Gatzlaff P, Konecke C, Hansen W, Schmitt-Thomsen A, Hertenstein B, Buer J, Ganser A (2003). G-CSF as immune regulator in T cells expressing the G-CSF receptor: implications for transplantation and autoimmune diseases. Blood.

[18] Zou L, Barnett B, Safah H, Larussa VF, Evdemon-Hogan M, Mottram P, Wei S, David O, Curiel TJ, Zou W (2004). Bone marrow is a reservoir for CD4+CD25+ regulatory T cells that traffic through CXCL12/CXCR4 signals. Cancer Res.

[19] Mielcarek M, Graf L, Johnson G, Torok-Storb B (1998). Production of interleukin-10 by granulocyte colony-stimulating factor-mobilized blood products: a mechanism for monocyte-mediated suppression of T-cell proliferation. Blood.

[20] Mielcarek M, Martin PJ, Torok-Storb B (1997). Suppression of alloantigen-induced T-cell proliferation by CD14+ cells derived from granulocyte colony-stimulating factor-mobilized peripheral blood mononuclear cells. Blood.

[21] Rodriguez PC, Zea AH, DeSalvo J, Culotta KS, Zabaleta J, Quiceno DG, Ochoa JB, Ochoa AC (2003). L-arginine consumption by macrophages modulates the expression of CD3 zeta chain in T lymphocytes. J Immunol.

[22] Arpinati M, Green CL, Heimfeld S, Heuser JE, Anasetti C (2000). Granulocyte-colony stimulating factor mobilizes T helper 2-inducing dendritic cells. Blood.

[23] Perobelli SM, Mercadante AC, Galvani RG, Goncalves-Silva T, Alves AP, Pereira-Neves A, Benchimol M, Nobrega A, Bonomo A (2016). G-CSF-induced suppressor IL-10+ neutrophils promote regulatory T cells that inhibit graft-versus-host disease in a long-lasting and specific way. J Immunol.

[24] Luyckx A, Schouppe E, Rutgeerts O, Lenaerts C, Fevery S, Devos T, Dierickx D, Waer M, Van Ginderachter JA, Billiau AD (2012). G-CSF stem cell mobilization in human donors induces polymorphonuclear and mononuclear myeloid-derived suppressor cells. Clin Immunol.

[25] Vendramin A, Gimondi S, Bermema A, Longoni P, Rizzitano S, Corradini P, Carniti C (2014). Graft monocytic myeloid-derived suppressor cell content predicts the risk of acute graft-versus-host disease after allogeneic transplantation of granulocyte colony-stimulating factor-mobilized peripheral blood stem cells. Biol Blood Marrow Transplant.

[26] Lv M, Zhao XS, Hu Y, Chang YJ, Zhao XY, Kong Y, Zhang XH, Xu LP, Liu KY, Huang XJ (2015). Monocytic and promyelocytic myeloid-derived suppressor cells may contribute to G-CSF-induced immune tolerance in haplo-identical allogeneic hematopoietic stem cell transplantation. Am J Hematol.

[27] Fan Q, Liu H, Liang X, Yang T, Fan Z, Huang F, Ling Y, Liao X, Xuan L, Xu N (2017). Superior GVHD-free, relapse-free survival for G-BM to G-PBSC grafts is associated with higher MDSCs content in allografting for patients with acute leukemia. J Hematol Oncol.

[28] Mougiakakos D, Jitschin R, von Bahr L, Poschke I, Gary R, Sundberg B, Gerbitz A, Ljungman P, Le Blanc K (2013). Immunosuppressive CD14+HLA-DRlow/neg IDO+ myeloid cells in patients following allogeneic hematopoietic stem cell transplantation. Leukemia.

[29] Solito S, Falisi E, Diaz-Montero CM, Doni A, Pinton L, Rosato A, Francescato S, Basso G, Zanovello P, Onicescu G (2011). A human promyelocytic-like population is responsible for the immune suppression mediated by myeloid-derived suppressor cells. Blood.

[30] Wang Y, Liu QF, Xu LP, Liu KY, Zhang XH, Ma X, Fan ZP, Wu DP, Huang XJ (2015). Haploidentical vs identical-sibling transplant for AML in remission: a multicenter, prospective study. Blood.

[31] Wang Y, Liu QF, Xu LP, Liu KY, Zhang XH, Ma X, Wu MQ, Wu DP, Huang XJ (2016). Haploidentical versus matched-sibling transplant in adults with Philadelphia-negative high-risk acute lymphoblastic leukemia: a biologically phase III randomized study. Clin Cancer Res.

[32] Wang Y, Wang HX, Lai YR, Sun ZM, Wu DP, Jiang M, Liu DH, Xu KL, Liu QF, Liu L (2016). Haploidentical transplant for myelodysplastic syndrome: registry-based comparison with identical sibling transplant. Leukemia.

[33] Xu LP, Liu KY, Liu DH, Han W, Chen H, Chen YH, Zhang XH, Wang Y, Wang FR, Wang JZ, Huang XJ (2012). A novel protocol for haploidentical hematopoietic SCT without in vitro T-cell depletion in the treatment of severe acquired aplastic anemia. Bone Marrow Transplant.

[34] Xu LP, Wang SQ, Wu DP, Wang JM, Gao SJ, Jiang M, Wang CB, Zhang X, Liu QF, Xia LH (2016). Haplo-identical transplantation for acquired severe aplastic anaemia in a multicentre prospective study. Br J Haematol.

[35] Armand P, Gibson CJ, Cutler C, Ho VT, Koreth J, Alyea EP, Ritz J, Sorror ML, Lee SJ, Deeg HJ (2012). A disease risk index for patients undergoing allogeneic stem cell transplantation. Blood.

[36] Przepiorka D, Weisdorf D, Martin P, Klingemann HG, Beatty P, Hows J, Thomas ED. 1994 Consensus Conference on Acute GVHD Grading. Bone Marrow Transplant. 1995(15):825–8. 7581076

[37] Filipovich AH, Weisdorf D, Pavletic S, Socie G, Wingard JR, Lee SJ, Martin P, Chien J, Przepiorka D, Couriel D (2005). National Institutes of Health consensus development project on criteria for clinical trials in chronic graft-versus-host disease: I. Diagnosis and staging working group report. Biol Blood Marrow Transplant.

[38] Fujii H, Luo ZJ, Kim HJ, Newbigging S, Gassas A, Keating A, Egeler RM (2015). Humanized chronic graft-versus-host disease in NOD-SCID il2rgamma-/- (NSG) mice with G-CSF-mobilized peripheral blood mononuclear cells following cyclophosphamide and total body irradiation. PLoS One.

[39] Walsh NC, Kenney LL, Jangalwe S, Aryee KE, Greiner DL, Brehm MA, Shultz LD (2017). Humanized mouse models of clinical disease. Annu Rev Pathol.

[40] Ferrara JL, Levy R, Chao NJ (1999). Pathophysiologic mechanisms of acute graft-vs.-host disease. Biol Blood Marrow Transplant.

[41] Krenger W, Hill GR, Ferrara JL (1997). Cytokine cascades in acute graft-versus-host disease. Transplantation.

[42] Gabrilovich DI, Ostrand-Rosenberg S, Bronte V (2012). Coordinated regulation of myeloid cells by tumours. Nat Rev Immunol.

[43] Kumar V, Patel S, Tcyganov E, Gabrilovich DI (2016). The nature of myeloid-derived suppressor cells in the tumor microenvironment. Trends Immunol.

[44] Solito S, Marigo I, Pinton L, Damuzzo V, Mandruzzato S, Bronte V (2014). Myeloid-derived suppressor cell heterogeneity in human cancers. Ann N Y Acad Sci.

[45] Srivastava MK, Andersson A, Zhu L, Harris-White M, Lee JM, Dubinett S, Sharma S (2012). Myeloid suppressor cells and immune modulation in lung cancer. Immunotherapy.

[46] Hamilton JA (2015). GM-CSF as a target in inflammatory/autoimmune disease: current evidence and future therapeutic potential. Expert Rev Clin Immunol.

[47] Barreda DR, Hanington PC, Belosevic M (2004). Regulation of myeloid development and function by colony stimulating factors. Dev Comp Immunol.

[48] Lechner MG, Liebertz DJ, Epstein AL (2010). Characterization of cytokine-induced myeloid-derived suppressor cells from normal human peripheral blood mononuclear cells. J Immunol.

[49] Marigo I, Bosio E, Solito S, Mesa C, Fernandez A, Dolcetti L, Ugel S, Sonda N, Bicciato S, Falisi E (2010). Tumor-induced tolerance and immune suppression depend on the C/EBPbeta transcription factor. Immunity.

[50] Krenger W, Snyder KM, Byon JC, Falzarano G, Ferrara JL (1995). Polarized type 2 alloreactive CD4+ and CD8+ donor T cells fail to induce experimental acute graft-versus-host disease. J Immunol.

[51] Hashimoto D, Asakura S, Miyake S, Yamamura T, Van Kaer L, Liu C, Tanimoto M, Teshima T (2005). Stimulation of host NKT cells by synthetic glycolipid regulates acute graft-versus-host disease by inducing Th2 polarization of donor T cells. J Immunol.

[52] Edinger M, Hoffmann P, Ermann J, Drago K, Fathman CG, Strober S, Negrin RS (2003). CD4+CD25+ regulatory T cells preserve graft-versus-tumor activity while inhibiting graft-versus-host disease after bone marrow transplantation. Nat Med.

[53] Li J, Heinrichs J, Haarberg K, Semple K, Veerapathran A, Liu C, Anasetti C, Yu XZ (2015). HY-specific induced regulatory T cells display high specificity and efficacy in the prevention of acute graft-versus-host disease. J Immunol.

[54] Heinrichs J, Li J, Nguyen H, Wu Y, Bastian D, Daethanasanmak A, Sofi MH, Schutt S, Liu C, Jin J (2016). CD8(+) Tregs promote GVHD prevention and overcome the impaired GVL effect mediated by CD4(+) Tregs in mice. Oncoimmunology.

[55] Ehx G, Somja J, Warnatz HJ, Ritacco C, Hannon M, Delens L, Fransolet G, Delvenne P, Muller J, Beguin Y (2018). Xenogeneic graft-versus-host disease in humanized NSG and NSG-HLA-A2/HHD mice. Front Immunol.

[56] Messmann JJ, Reisser T, Leithauser F, Lutz MB, Debatin KM, Strauss G (2015). In vitro-generated MDSCs prevent murine GVHD by inducing type 2 T cells without disabling antitumor cytotoxicity. Blood.

