# Supplementary material for: Early myeloid-derived suppressor cells (HLA-DR−/lowCD33+CD16−) expanded by granulocyte colony-stimulating factor prevent acute graft-versus-host disease (GVHD) in humanized mouse and might contribute to lower GVHD in patients post allo-HSCT
Source: J Hematol Oncol. 2019 Mar 18;12:31. doi: 10.1186/s13045-019-0710-0 (PMC6423891; doi:10.1186/s13045-019-0710-0)
Supplement: Supplementary file 1 — Table S1. Cell components in graft. Figure S1. HLA-DR−/lowCD33+CD16− eMDSCs demonstrated superior immune-suppressive activity compared with CD33− and HLA-DR−/lowCD33+CD16+ fraction. Figure S2. Comparison of immune-suppressive activity of HLA-DR−/lowCD33+CD16− eMDSCs with M-MDSCs and G-MDSCs. Figure S3. Selective inhibitors of arginase (nor-NOHA), iNOS (L-NMMA), and IDO (NLG8189) on T cell proliferation. Figure S4. Human white blood cell engraftment at 7 days, 14 days, and 21 days after co-transplantation. Figure S5. eMDSC engraftment in NSG mice at 14 and 21 days after co-transplantation. Figure S6. Treg, Th1, Th2, and Th17 cells detected in the peripheral blood in NSG mice at day 21. Figure S7. Cytokines detected in the peripheral blood of NSG mice at 7, 14, and 21 days after co-transplantation. (DOCX 724 kb) [file 13045_2019_710_MOESM1_ESM.docx]

| G-BM+ G-PBSC Graft | Mean and range(10^8/kg) |
| --- | --- |
| leukocyte | 11.24(7.14-21.42) |
| CD34+ | 2.89(0.62-6.85) |
| Lymphocyte | 3.73(0.40-8.47) |
| CD14+ Monocyte | 1.83(0.67-4.19) |
| CD3+ T cells | 2.60(0.61-5.79) |
| CD4+ T cells | 1.41(0.32-3.42) |
| CD8+ T cells | 0.84(0.15-2.29) |
| eMDSC | 1.98(0.52-7.01) |

**Table S1** **Cell components in Graft**

Abbreviations: G-BM, G-CSF primed bone marrow; G-PBSC, G-CSF peripheral blood stem cells harvest;


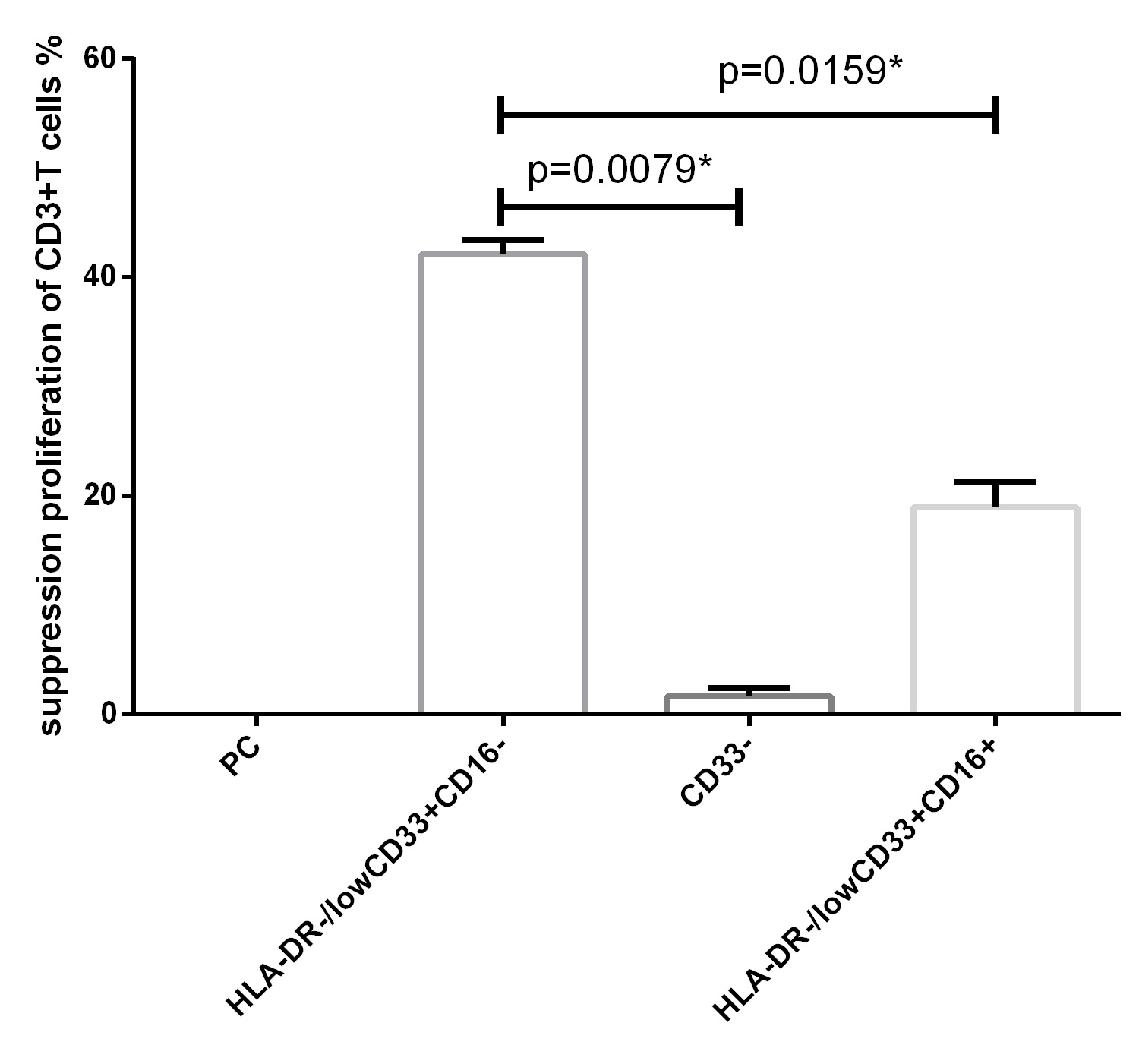


**Figure S1 HLA-DR^-/low^CD33^+^CD16^-^ eMDSCs demonstrated superior immune-suppressive activity compared with CD33^-^ and HLA-DR^-/low^CD33^+^CD16^+^ fraction**

PC, positive control; * Statistical Significance


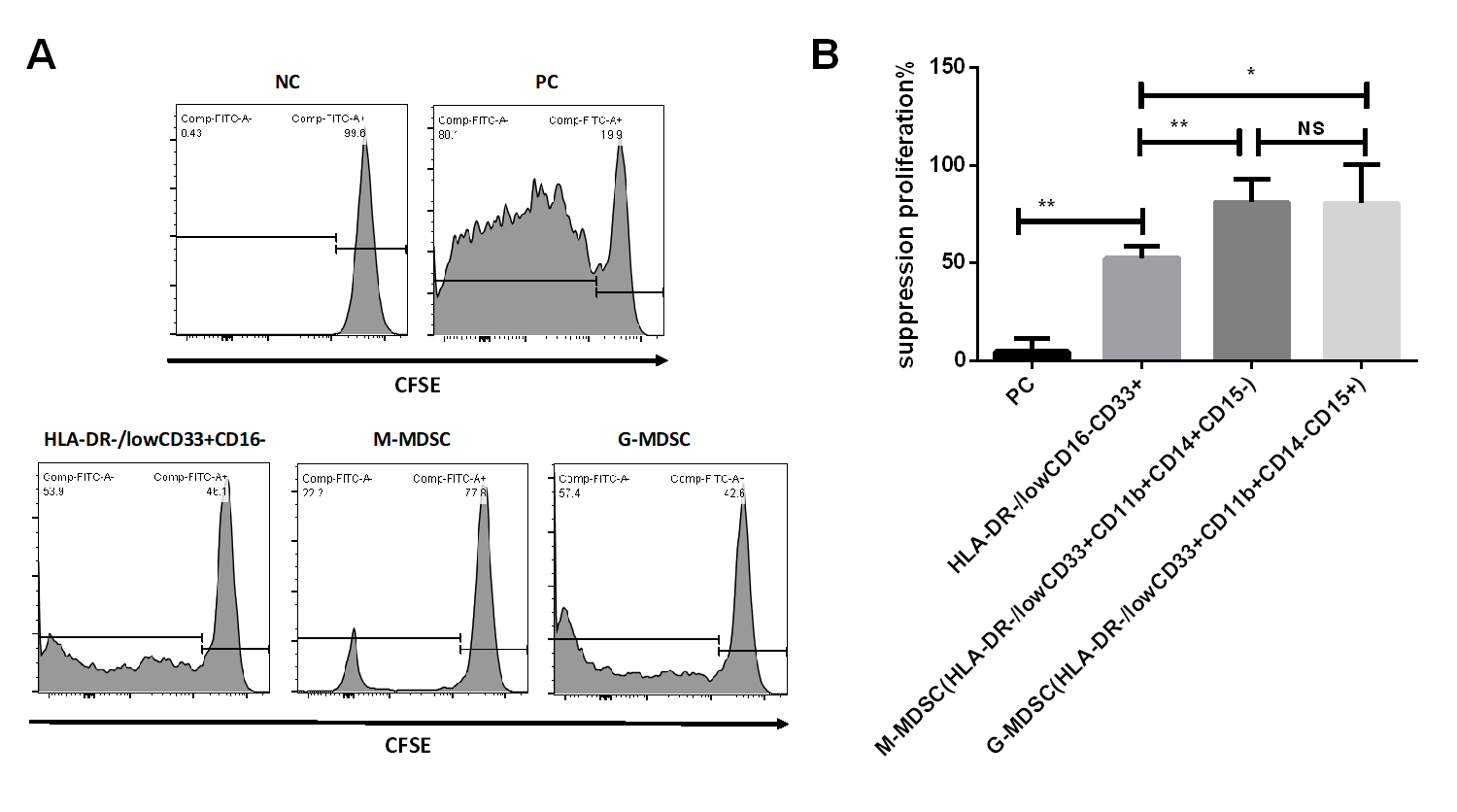


**Figure S2 Comparison of immune-suppressive activity of HLA-DR^-/low^CD33^+^CD16^-^ eMDSCs with M-MDSCs and G-MDSCs**

* Statistical Significance P<0.05; ** Statistical Significance P<0.01

PC, positive control; NC, negative control

**Figure S3 Selective inhibitors of arginase(nor-NOHA), iNOS (L-NMMA), IDO (NLG8189) on T cell proliferation**

PC, positive control; MDSC, HLA-DR^-/low^CD33^+^CD16^-^ eMDSCs





**Figure S4 Human white blood cell engraftment at 7 days, 14 days and 21 days after co-transplantation.** A. the percentage of human leukocytes in whole blood cells collected from transplanted mice. B. No significant difference of human cells engraftment intragroup





**Figure S5 eMDSC engraftment in NSG mice at 14 and 21 days after** **co-transplantation.**

1. Gating strategies of human HLA-DR^-/low^CD33^+^CD16^-^MDSC. B. MDSC engraftment in peripheral blood(PB), spleen(SP) and bone marrow (BM) in NSG mice at 14 days after transplantation and 21 days after transplantation.

**

**

**Figure S6 Treg, Th1, Th2 and Th17 cells detected in peripheral blood in NSG mice at days 21.**

A. percentage of Treg(CD4^+^CD25^+^Foxp3^+^) in peripheral blood.

B. percentage of Th1(CD4^+^T-bet^+^), Th2(CD4^+^GATA3^+^) and Th17(CD4^+^RORγt^+^) in peripheral blood.

.
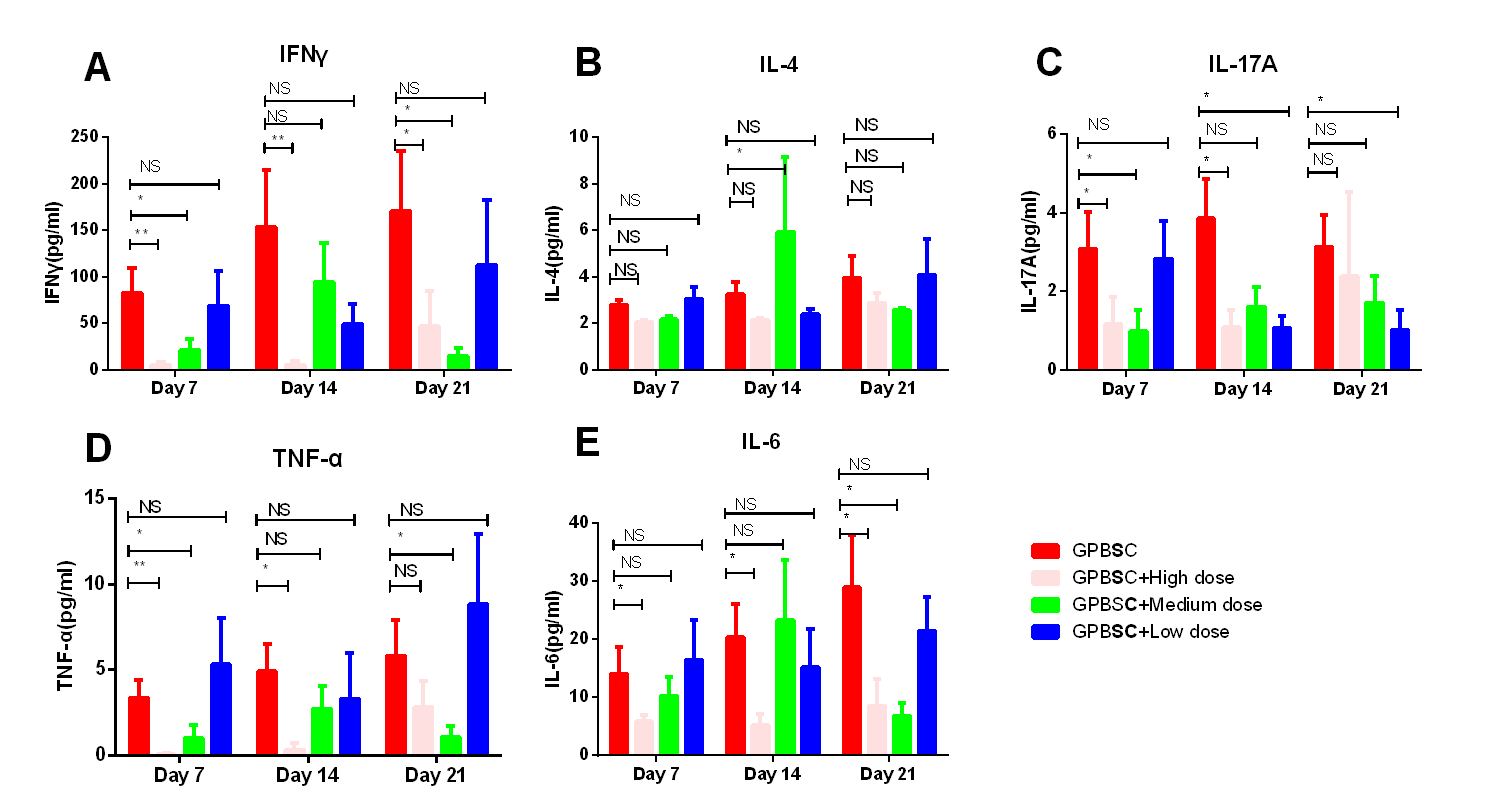


**Figure S7 Cytokines detected in peripheral blood of NSG mice at 7, 14 and 21 days after** **co-transplantation.**

1. INF-γ; B. IL-4; C IL-17A; D TNF-α; E IL-6

* Statistical Significance P<0.05; ** Statistical Significance P<0.01
